# Atomic species identification at the (101) anatase surface by simultaneous scanning tunnelling and atomic force microscopy

**DOI:** 10.1038/ncomms8265

**Published:** 2015-06-29

**Authors:** Oleksandr Stetsovych, Milica Todorović, Tomoko K. Shimizu, César Moreno, James William Ryan, Carmen Pérez León, Keisuke Sagisaka, Emilio Palomares, Vladimír Matolín, Daisuke Fujita, Ruben Perez, Oscar Custance

**Affiliations:** 1 National Institute for Materials Science (NIMS), 1-2-1 Sengen, Tsukuba 305-0047, Japan; 2 Charles University, Faculty of Mathematics and Physics, V Holešovičkách 2, Praha 8, Czech Republic; 3 Departamento de Física Teórica de la Materia Condensada, Universidad Autónoma de Madrid, Madrid 28049, Spain; 4 JST, PRESTO, 4-1-8 Honcho, Kawaguchi, Saitama 332-0012, Japan; 5 International Center for Young Scientists, NIMS, 1-2-1 Sengen, Tsukuba 305-0047, Japan; 6 Catalan Institute of Nanoscience and Nanotechnology (ICN2), Bellaterra, Barcelona 08193, Spain; 7 Karlsruhe Institute of Technology (KIT), Wolfgang Gaede Str. 1, Karlsruhe 76131, Germany; 8 Institute of Chemical Research of Catalonia, Av. Països Catalans 16, Tarragona 43007, Spain; 9 ICREA, Passeig Lluís Companys 23, Barcelona 08010, Spain; 10 Condensed Matter Physics Center (IFIMAC), Universidad Autónoma de Madrid, Madrid 28049, Spain

## Abstract

Anatase is a pivotal material in devices for energy-harvesting applications and catalysis. Methods for the accurate characterization of this reducible oxide at the atomic scale are critical in the exploration of outstanding properties for technological developments. Here we combine atomic force microscopy (AFM) and scanning tunnelling microscopy (STM), supported by first-principles calculations, for the simultaneous imaging and unambiguous identification of atomic species at the (101) anatase surface. We demonstrate that dynamic AFM-STM operation allows atomic resolution imaging within the material's band gap. Based on key distinguishing features extracted from calculations and experiments, we identify candidates for the most common surface defects. Our results pave the way for the understanding of surface processes, like adsorption of metal dopants and photoactive molecules, that are fundamental for the catalytic and photovoltaic applications of anatase, and demonstrate the potential of dynamic AFM-STM for the characterization of wide band gap materials.

## Abstract (web summary)

Anatase is a pivotal material in devices for energy-harvesting applications and catalysis. Here, Stetsovych *et al*. demonstrate the potential of simultaneously combining atomic force microscopy and scanning tunnelling microscopy to identify the atomic species populating the (101) surface of anatase.

## Graphical abstract

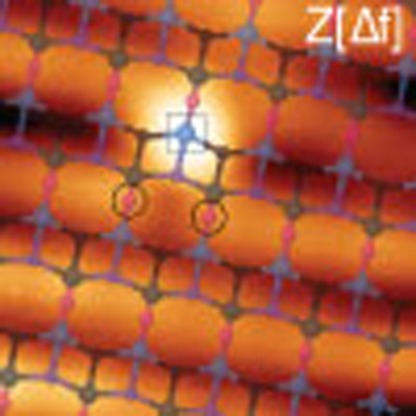

Titanium dioxide (TiO_2_) is an important material in a number of energy-related applications such as photocatalytic water splitting^1^,^2^ and the conversion of solar energy to electricity^3^,^4^. This material is also used for surface anticorrosion, water purification and decomposition of organic pollutants^5^,^6^. Most of these applications rely on nanocrystalline TiO_2_ samples that consist principally of two polymorphs: anatase and rutile. In some commercial TiO_2_ samples, anatase nano-crystals account for up to 75% of the product^7^. Anatase is generally regarded as having a higher surface reactivity than rutile and, for nano particles with diameters <11 nm, it is more stable than rutile^5^. In contrast to the performance of rutile, the extraordinary mobility and low recombination rate of photo-induced charges found in anatase accounts for a high-power conversion efficiency in solar cells^8^ and a considerable enhancement of the photocatalytic production of hydrogen^9^. Considering that anatase nano-crystals are the more abundant and reactive components in TiO_2_ samples used as the active phase in commercial catalysts, there is still a relatively scarce amount of experimental studies on anatase surfaces in comparison with rutile. More research is required to better understand the surface properties that define anatase as such a good photocatalyst. The real space characterization of anatase substrates at the atomic scale and, in particular, the understanding of the structure of the common defects and their role in the surface chemistry and charge transport properties, is essential to elucidate the basic principles that govern photocatalytic and photovoltaic applications of this TiO_2_ polymorph.

Transmission electron microscopy can provide atomic scale images of micro- and nano-scale anatase crystals^10^ even close to photocatalytic reaction conditions^11^, while scanning tunnelling microscopy^12^ (STM) is the common choice to study the atomic structure^13^,^14^ and defects^15^,^16^ of anatase surfaces, as well as the properties and behaviour of adsorbates^17^,^18^,^19^,^20^. Atomic force microscopy^21^ (AFM) has recently also shown considerable potential to study the properties of rutile surfaces with atomic resolution^22^,^23^,^24^,^25^,^26^,^27^,^28^,^29^. While the phenomenology of rutile has been extensively studied at atomic scale over several decades with both STM and AFM, the amount of information regarding the properties of anatase surfaces is still scarce. At a very fundamental level, there is still debate regarding the contribution of the different atoms populating the anatase surface to the STM images^13^,^30^. There is a clear need for further experimental insight to support STM-based observations on anatase, and yet there have been no atomic resolution AFM studies to date.

In this work, we simultaneously apply dynamic AFM and STM (see Methods) to study anatase (101); the energetically most stable facet of this TiO_2_ polymorph. Such a combination of techniques, together with the use of individual water molecules as atomic markers, provides a clear way to experimentally identify the atomic species populating this surface: while AFM images the topmost oxygen atoms, the main contribution to the averaged tunnelling current (the STM signal in our experiments) comes from the titanium atoms at the third atomic layer. First-principles simulations of the tip–surface interaction confirm this identification, present a plausible description of the forefront part of the experimental probe and reveal the role of atomic relaxation effects in the AFM contrast formation. From our simulations of realistic surface detects, we extract dominant attributes to identify candidates of subsurface oxygen vacancies and surface hydroxyls from an extensive collection of dynamic AFM-STM images acquired over multiple measurement sessions.

## Results

### Simultaneous dynamic AFM-STM imaging

**Figure 1:**
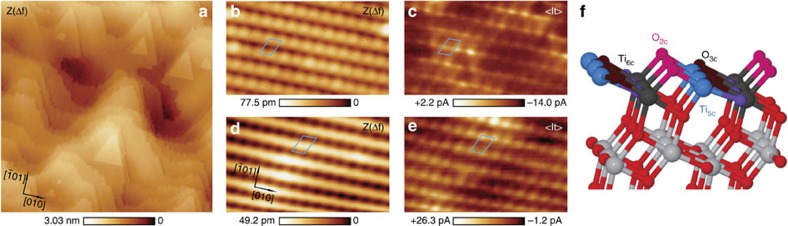
Simultaneous AFM and averaged tunnelling current images of the TiO_2_(101) anatase surface. (**a**) AFM topographic image representing the general morphology of the surface over a (160 × 160) nm^2^ area. Acquisition parameters (see Methods) are: *f*_o_=170,999 Hz; Δ*f*=−6.5 Hz; *A*=261.3 Å; *K*=33.4 N m^−1^; CPD=*V*_Bias_=−243 mV. (**b**,**c**) Simultaneous topographic AFM (*Z*(Δ*f*)) and averaged tunnelling current (<I_*t*_>) data showing a characteristic atomic pattern that displays well-defined ovals along the rows of protrusions appearing in the *Z*(Δ*f*) image. Acquisition parameters are: *f*_o_=153,031 Hz; Δ*f*=−47.4 Hz; *A*=107.1 Å; *K*=23.9 N m^−1^; CPD=*V*_Bias_=+800 mV. (**d**,**e**) Simultaneous *Z*(Δ*f*) and <I_*t*_> images obtained with a different tip termination, and corresponding to a less frequent atomic pattern characterized by featureless rows of protrusions in the *Z*(Δ*f*) image. Acquisition parameters are: *f*_o_=158,957 Hz; Δ*f*=−6.0 Hz; *A*=143.2 Å; *K*=26.8 N m^−1^; CPD=*V*_Bias_=+510 mV. For both sets, image dimensions are (5 × 3) nm^2^. The parallelogram marks the same surface area in **b**,**c** and **d**,**e**, respectively. The crystallographic directions of the surface are indicated in **a** and **d**. All the experimental images in this work display identical orientation with respect to these directions. (**f**) Ball-and-stick model of the TiO_2_(101) anatase surface, which terminates in rows of twofold coordinated oxygen atoms (O_2*c*_), followed by a bilayer of threefold coordinated oxygen (O_3*c*_, second atomic layer) and fivefold coordinated titanium (Ti_5*c*_, third atomic layer) atoms, and a deeper second bilayer of O_3*c*_ and sixfold coordinated titanium (Ti_6*c*_) atoms.

Figure 1a shows the general morphology of the TiO_2_(101) anatase surface measured with AFM. Characteristic triangular and truncated trapezoidal terraces and islands are clearly observed, in good agreement with previous STM results^14^. Typical atomic scale AFM and averaged tunnelling current^31^ (<I_*t*_>) images are characterized by rows of signal maxima running along the [010] crystallographic direction (Fig. 1b–e). We have identified two typical atomic patterns in AFM images: pattern A (Fig. 1b) displaying clear ovals along the rows of protrusions; and pattern B (Fig. 1d) showing almost featureless bright rows, and accounting for ∼10% incidence. The variability for the <I_*t*_> images is wider (see also Supplementary Figs 1–3). Apart from the inherent dependence on the tip–surface separation and bias voltage, the latter points towards a marked dependence of the <I_*t*_> signal on the nature of the probe termination. The comparison of simultaneous AFM and <I_*t*_> images reveals that the bright features corresponding to high current signal are mainly located in between the rows of protrusions detected by AFM.

The atomic structure of the TiO_2_(101) anatase surface is represented in Fig. 1f. Rows of twofold coordinated oxygen (O_2*c*_) atoms form the topmost part of the surface. Below, there are two oxygen-titanium bilayers in which the oxygen atoms are threefold coordinated (O_3*c*_), and the titanium atoms are five (Ti_5*c*_) and sixfold coordinated (Ti_6*c*_) at the shallower and deeper bilayers, respectively. On this complex surface structure, there is not a univocal way to match the positions of the different atomic species to the maxima observed in the atomic resolution AFM and <I_*t*_> images shown in Fig. 1b–e.

### Identification of atomic species

**Figure 2:**
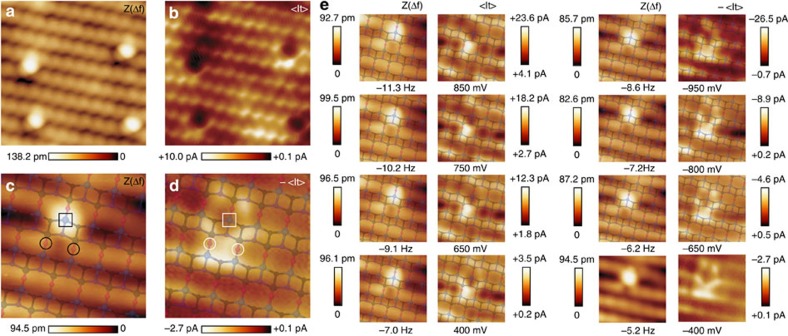
Individual water molecules as atomic markers. (**a**,**b**) Simultaneous topographic AFM (*Z*(Δ*f*)) and averaged tunnelling current (<I_*t*_>) images showing four individual water molecules adsorbed on the TiO_2_(101) anatase surface. Image dimensions are (4.5 × 4.5) nm^2^. Simultaneous *Z*(Δ*f*) (**c**) and <I_*t*_> (**d**) signals ascribed to a single water molecule imaged with a different—more symmetric—tip termination. A top view of the outer atomic layers of the TiO_2_(101) anatase surface has been superimposed to the images (see text for details). The squares mark the Ti_5*c*_ atom at which the water molecule binds to the surface^17^. The circles highlight the O_2*c*_ atoms that sustain two weak hydrogen bonds with the water molecule^17^. (**e**) Sets of simultaneous *Z*(Δ*f*) and <I_*t*_> bias-dependent images obtained over the water molecule displayed in **c** and **d** with identical tip termination and approximately keeping the same tip–surface separation (see Methods). The contrast of the filled state images (negative sample bias voltage) has been inverted (−<I_*t*_>) for a better comparison with the empty state data. These images without the atomic model of the anatase (101) surface superimposed are displayed in Supplementary Fig. 4. **c** and **d** are a magnification of the images labelled as −400 mV in **e**. Image dimensions are (2 × 2) nm^2^. Acquisition parameters are: *f*_o_=159,989 Hz, Δ*f*=−6.6 Hz, A=118.0 Å, *K*=27.3 N m^−1^, CPD=*V*_Bias_=+400 mV, for **a** and **b**; and *f*_o_=159,989 Hz, *A*=113.2 Å, *K*=27.3 N m^−1^, CPD=−180 mV for **c**–**e**. The Δ*f* set point and the *V*_Bias_ value are listed under each set of images in **e**.

In early STM studies on anatase surfaces, the assignment of the imaged atomic species was normally presumed following the assumption that at sample bias voltages close to the conduction band, the Ti atoms should contribute the most to the tunnelling current, by analogy with the case of the TiO_2_(110) rutile surface^32^. However, the contribution of the surface atoms to the topographic STM images is subject to the current set point and the bias voltage^17^. In this work, to experimentally verify the contribution of the surface atomic species to our AFM and <I_*t*_> images, we use individual water molecules—intentionally deposited on the surface—as atomic markers (Fig. 2). Previous theoretical works have shown that the oxygen atom of a water molecule adsorbed on anatase (101) strongly binds to one of the Ti_5*c*_ atoms^17^. This bond produces a redistribution of the local density of the states around the targeted Ti_5*c*_ atom that makes it vanish from an STM image^17^. The water molecule additionally sustains two weak hydrogen bonds with O_2*c*_ atoms at the closest oxygen atomic row^17^. These bonding features are clearly identified in our atomic resolution images.

Figure 2a,b displays simultaneous AFM and <I_*t*_> images of four individual water molecules adsorbed on the TiO_2_(101) anatase surface. In the topographic AFM images, a water molecule appears as a protrusion between two rows of ovals, elevated 55±6 pm from them. All the molecules adsorb in the same orientation that is denoted by the presence of a dip at one of the nearby rows. The <I_*t*_> channel shows a depletion of the current signal at the same location the water molecule is imaged by AFM. The details of the features ascribed to the adsorption of a water molecule imaged with a more symmetric AFM tip termination become apparent in Fig. 2c,d, which are a magnification of the images labelled as −400 mV in Fig. 2e. Considering previous theoretical predictions^17^, it is possible to superimpose a model of the five shallower atomic layers of the TiO_2_(101) anatase structure to these images by assigning a Ti_5*c*_ site at the maximum topographic signal associated with the water molecule, and by aligning the atomic rows of the model with the rows of ovals in the AFM image. This procedure always results in the O_2*c*_ atoms lying on top of the protruding ovals detected by AFM. A 180° rotation of the atomic model is excluded due to the asymmetry imposed by energetically inequivalent surface steps^14^. The comparison of the surface model with the features shown in the atomic resolution images of Fig. 2c,d suggests that AFM is sensitive to the topmost atomic layer of the surface, imaging the O_2*c*_ atoms as protrusions, while the main contribution to the tunnelling current is related to the Ti_5*c*_ atoms.

This assignment of the different atomic species imaged by AFM and <I_*t*_> clarifies the features observed for the adsorption of molecular water on the TiO_2_(101) anatase surface. The dip between the atoms highlighted with circles in Fig. 2c is the signature of the hydrogen bonds of the water molecule with the two O_2*c*_ atoms^17^. In the corresponding <I_*t*_> image (Fig. 2d), the Ti_5*c*_ atom binding to the water molecule—highlighted by a square—has no apparent contribution to the tunnelling current. Instead, the signal appears redistributed in a region close to the O_2*c*_ atoms involved in the hydrogen bonds, the Ti_5*c*_ atoms interacting with these two O_2*c*_ atoms and two additional lobes near the O_2*c*_ that binds to the highlighted Ti_5*c*_ atom. Most of the features observed in these <I_*t*_> images are in good agreement with simulated STM data based on our first-principles calculations and with previous theoretical works^17^. Our simulations confirm the experimental observations that Ti_5*c*_ surface sites present as STM maxima when imaging at positive sample bias voltages. The connection between our <I_*t*_> data and conventional topographic STM images is discussed below.

### Bias-dependent imaging

Dynamic AFM-STM not only provides a clear identification of the atomic species responsible for the image contrast but paves the way for atomic resolution imaging at relatively small positive and negative bias voltages, in contrast with previously reported STM measurements on anatase. Figure 2e summarises a set of simultaneous AFM and <I_*t*_> bias-dependent images—keeping the tip–surface separation approximately constant (see Methods)—measured above the water molecule displayed in Fig. 2c,d. These images were acquired with identical probe termination over a bias voltage range from −820 mV to +950 mV, having a tip–surface contact potential difference of −180 mV. The AFM data present the same atomic features regardless of the applied bias, showing an expected lower contrast for higher bias voltages due to an increasing contribution of the long-range electrostatic force. The <I_*t*_> empty and filled state images slightly differ, yet both indicate localization of density of states over the same surface region, as well as depletion of charge from the Ti_5*c*_ atom binding the water molecule. These <I_*t*_> images show a negligible variation of the local density of states on the bias voltage range explored in the experiment. Even a more striking fact is the collection of an apparent tunnelling current within the band gap of the TiO_2_(101) anatase surface, that has the onset of the conduction band located between +0.5 V and +0.75 V as reported by scanning tunnelling spectroscopy measurements^33^,^34^. In static STM topographic measurements performed without cantilever oscillation (Supplementary Fig. 2), stable empty state imaging was achieved by setting relatively high bias voltages (typically between +0.8 V to +2 V depending on the tip condition), while stable filled state imaging was almost impossible even for biases as large as −2 V. Although the mechanism for the acquisition of current signal within the surface band gap is not clear yet (it may be related to either the existence of defect states within the band gap of our anatase natural single crystals or band-bending effects^35^), these results show how, for surface systems where the AFM and the tunnelling current signals are spatially decoupled (see Supplementary Fig. 3), simultaneous dynamic AFM-STM bears enormous potential to study surfaces of wide band gap materials close to the Fermi level with atomic resolution.

## Discussion

**Figure 3:**
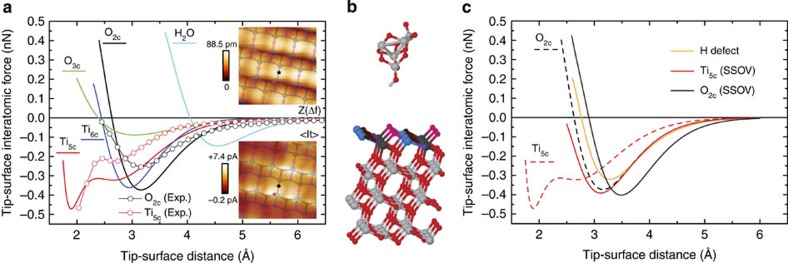
AFM imaging contrast mechanism and common surface defects. (**a**) Calculated tip–surface interatomic forces over the relevant atomic positions of the TiO_2_(101) anatase surface and comparison with experimental curves. The insets are simultaneous topographic AFM (*Z*(Δ*f*), upper panel) and averaged tunnelling current (<I_*t*_>, lower panel) images of the surface area where the force spectroscopy experiments were performed. The black and red stars mark the acquisition spots. Image dimensions are (1.5 × 1.5) nm^2^. Acquisition parameters are: *f*_o_=160,360 Hz, Δ*f*=−5.0 Hz, *A*=113.6 Å, *K*=27.5 N m^−1^, CPD=−80 mV, *V*_Bias_=+500 mV. (**b**) Atomic model used in first-principle calculations, showing the hydroxyl group-terminated sharp TiO_2_ cluster tip above the the TiO_2_(101) anatase surface slab. (**c**) Calculated force spectroscopy curves at the relevant sites of common TiO_2_(101) surface point defects: a hydrogen defect and a subsurface oxygen vacancy (SSOV)—results for the clean surface sites are provided in dashed line for comparison.

The AFM imaging mechanism on the TiO_2_(101) anatase surface can be further clarified with the aid of first-principles calculations. Determining a suitable atomic arrangement that qualitatively describes the forefront part of the probe is crucial for the correct interpretation of AFM data^36^,^37^,^38^,^39^,^40^,^41^. To model the tip apex, we have chosen small TiO_2_ clusters terminated by a hydroxyl group, which were found to successfully describe weak tip–surface interatomic forces on TiO_2_(110) rutile^39^ and could have been easily formed during the probe conditioning prior to starting the measurements (see Methods). Calculated tip–surface interatomic forces obtained on approaching the probe model over relevant atomic positions of the TiO_2_(101) anatase surface were compared with the experimental counterparts. Both the magnitude of the force minima and the overall shape of the curves were evaluated for different relative orientations of the tip cluster model, exposing blunt or sharp cluster edges towards the surface, while maintaining the hydroxyl termination. Figure 3a summarises the calculated tip–surface interatomic forces obtained with the sharp probe orientation that best reproduces experimental data—depicted in Fig. 3b—and displays the comparison with a typical set of experimental short-range force curves.

**Figure 4:**
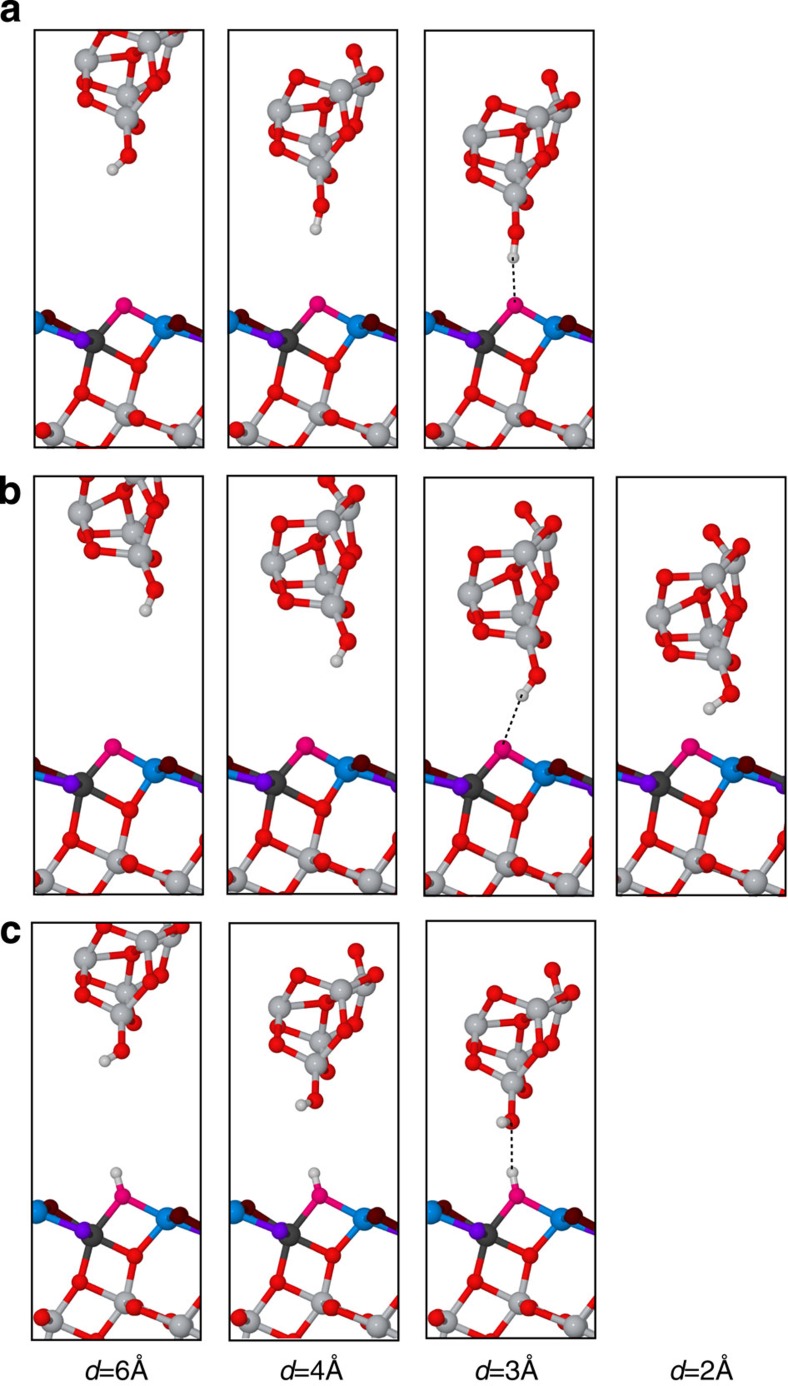
Atomic relaxations at the forefront of the tip model on interaction with the surface. Computational snapshots illustrating the evolution in optimal atomistic arrangement of tip and surface atoms as the tip model is approached towards: (**a**) an O_2*c*_ atomic site; (**b**) an Ti_5*c*_ atomic site, and (**c**) a hydroxyl defect site of the TiO_2_(101) anatase surface. Tip height (*d*) labels below the images are directly related to the tip–surface distance in computed force spectroscopy graphs shown in Fig. 3a,c. At *d*=3 Å, the relevant hydrogen bond interaction is highlighted with a dashed line. At *d*=2 Å, the images where the only system change is a small downwards shift of surface atoms due to tip–surface repulsion are omitted.

The analysis of the calculated forces corroborates the experimental observations and confirms that, at the onset of the tip–surface interatomic forces, AFM should image the water molecule and the O_2*c*_ atoms as protrusions. Atomic relaxations of the tip model at the O_2*c*_ site demonstrate that hydrogen bond formation between the hydroxyl group at the AFM probe and the surface oxygen atom dominates the interaction (Fig. 4a). In the same tip–surface separation regime, such atomic relaxations were also observed with tips probing the close-by Ti_6*c*_ and Ti_5*c*_ sites: the hydroxyl group at the probe re-orients towards the nearest O_2*c*_ site and a hydrogen bond is formed (Fig. 4b). Such tip relaxation effects explain the most common topographic AFM images (Fig. 1b) that exhibit rows of protruding ovals slightly elongated along the 
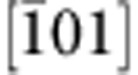
 crystallographic direction. Placing the tip model into a lower height regime reveals a double minima feature in the force curve over the Ti_5*c*_ site. This feature is due to the deflection of the tip hydrogen (H) atom at the first force minimum that further allows the exposed oxygen of the hydroxyl to engage in bonding with the sampled Ti_5*c*_ atom at the second minimum (Fig. 4b, *d*=2 Å). Traces of double force minima at Ti_5*c*_ sites can also be observed experimentally for some probes, as displayed in Fig. 3a. The O_3*c*_ surface atoms are laterally positioned furthest away from the O_2*c*_ sites, so the force curves computed over them are free of the hydrogen bond interaction with the O_2*c*_ atoms. The small forces predicted over the shallower O_3*c*_ atoms should make them almost undetectable by the AFM. Force spectroscopy at most atomic sites of the clean anatase surface—excluding the O_3*c*_ atoms—results in the tip hydroxyl group reorienting towards the nearest O_2*c*_ site, which leads to repeated sampling of the same chemical interaction and an extended, oval-shape maxima in the experimental AFM images.

**Figure 5:**
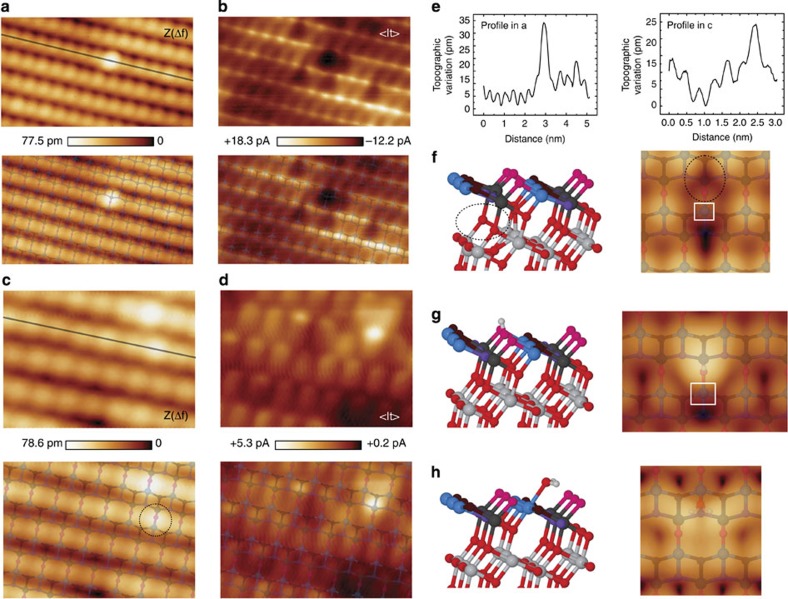
Identification of surface defects at the TiO_2_(101) anatase surface. (**a**) Simultaneous AFM topographic (*Z*(Δ*f*)) and (**b**) averaged tunnelling current (<I_*t*_>) images showing a candidate for a subsurface oxygen vacancy defect. Images of a defect candidate to represent a surface hydroxyl group are displayed on panels (**c**,**d**). Duplicates of these experimental images with a superimposed model of the outer atomic layers of the TiO_2_(101) anatase surface are also shown. (**e**) Variation of the topographic signal along the line profiles in **a** and **c**. The combination of these experimental images and our theoretical predictions provides the necessary clues for the identification of these atomic defects (see text). Acquisition parameters are: *f*_o_=153,031 Hz, Δ*f*=−50.0 Hz, *A*=107.1 Å, *K*=23.9 N·m^−1^, CPD=+800 mV, *V*_Bias_=+1,000 mV, for **a** and **b**; and *f*_o_=159,989 Hz, Δ*f*=−11.5 Hz, A=109.9 Å, K=27.3 N m^−1^, CPD=−180 mV, *V*_Bias_=+550 mV, for **c**–**e**. Image dimensions for **a** and **d** are (5.0 × 3) nm^2^ and (3.0 × 2.4) nm^2^, respectively. Atomic models of optimal geometries and corresponding Tersoff–Hamann STM images for: (**f**) a subsurface oxygen vacancy (0.8* *V); (**g**) a surface hydroxyl group (0.6 V) and (**h**) a water molecule attached to the surface (0.4 V). Dark areas in computed STM images in **f** and **g** appear near reduced 
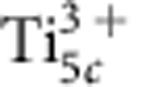
 surface sites (white squares) associated with the defect formation. The dotted-line ellipse in **f** highlights the position of the subsurface oxygen vacancy. An atomic model of the surface has been superimposed to the calculated STM images.

Identification of adsorbates on the anatase (101) surface using STM manipulation techniques have been recently demonstrated^42^. The combination of dynamic AFM-STM experiments and quantum mechanical simulations supplies alternative means for the identification of point defects on the anatase (101) surface. Using first-principles calculations, we have analyzed the structures of common defects expected at the anatase surface, such as subsurface oxygen vacancies^43^ and hydroxyl groups, and identified the key features they should exhibit in the experimental images (Fig. 5).

Subsurface oxygen vacancy sites alter both the surface geometry and the local electronic properties by inducing notable structural deformations and a localized surface defect state. By virtue of subsurface bond distortions, the O_2*c*_ site above the vacancy is lifted up by 28 pm while other surface atoms move downwards. This topographic change results in a rigid upward shift of the O_2*c*_ force curve of ∼30 pm for spectroscopy calculated on the O_2*c*_ atom above a subsurface oxygen vacancy (see Fig. 3c). One of the electrons left behind after the vacancy creation is localized at the nearest Ti_5*c*_ site next to the protruding O_2*c*_ (see square in Fig. 5f), that becomes a 
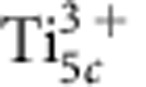
. This reduction process increases the reactivity with the probe and leads to an interaction force over the 
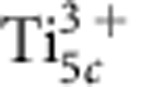
 atom that resembles the one for the O_2*c*_ atoms away from the defect (Fig. 3c). Consequently, the presence of a subsurface oxygen vacancy causes the adjacent O_2*c*_ atom to appear as a wide primary maximum in experimental AFM images with features extended over the 
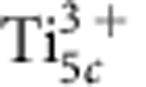
 atom nearby. At variance with the on-top bright feature expected for the AFM channel, our calculations predict a decrease of the STM signal at the 
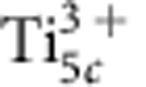
 site (Fig. 5f), which now hosts a band gap defect state that depletes the density of states in the conduction band responsible for the bright rows of STM maxima.

The defect shown in Fig. 5a,b features the above predicted contrast, and so is a candidate for a subsurface oxygen vacancy imaged with dynamic AFM-STM. The bright, wide feature in the topographic AFM image (Fig. 5a) is located at a O_2*c*_ site, and protrudes ∼28 pm from other O_2*c*_ atoms in defect-free regions of the surface (Fig. 5e), in excellent agreement with the calculations. This identification in the AFM image is further supported by the associated <I_*t*_> signal (Fig. 5b) measured at positive bias, which shows the main feature predicted by the calculated STM image (Fig. 3e): depletion of tunnelling current under the protrusion seen in the AFM topography.

Combined information from dynamic AFM-STM experiments also helps us identify a strong candidate for the subtle surface hydroxyl defect (Fig. 5c,d). Our calculations show that a hydrogen atom preferentially bonds to O_2*c*_ sites, resulting in the host atom moving 7 pm upwards. The surface hydroxyl interacts with the hydroxyl group at the probe model to deflect the H atom at the tip and to form a hydrogen bond with the exposed oxygen atom, thus mirroring the probe–surface interaction behind the imaging of the O_2*c*_ sites at the clean surface. The modest structural change and a similar interaction strength lead to a close correlation of simulated force minima over a hydrogen defect and an O_2*c*_ site, as shown in Fig. 3c. Accordingly, hydrogen defects on the TiO_2_(101) anatase surface should be almost indistinguishable from ordinary O_2*c*_ sites in AFM images collected with hydroxyl-terminated probes at the onset of the tip–surface interatomic force. However, computed STM images (Fig. 5g) suggest that this defect should be detectable in the averaged tunnelling current channel at positive bias, with the hydrogen marked by an extended triangular shape maximum between Ti_5*c*_ atomic rows.

These theoretical predictions match very well with the features observed for a defect shown in the dynamic AFM-STM images displayed in Fig. 5c,d. Along the line profile highlighted in Fig. 5c, there is a defect on a O_2*c*_ site protruding ∼11 pm with respect to other O_2*c*_ atoms located in less defective surface areas (see Fig. 5e). This weak contrast in the AFM topography, although compatible with the calculations, is clearly not enough for the identification. However, the appearance of the <I_*t*_> signal near this defect resembles the STM image predicted from our first-principles calculations for a surface hydroxyl group, with a bright triangular-shape protrusion between Ti_5*c*_ rows. These combined features provide strong support to identify the defect highlighted in Fig. 5c,d as a clear candidate for a surface hydroxyl group.

Notice that there are other surface defects visible in the <I_*t*_> images displayed in Fig. 5, yet they are barely detected in the AFM topography. Such defects may correspond to the presence of interstitial atoms^15^ or hydrogen incorporated into the bulk^44^. We have also observed variability of the <I_*t*_> background contrast over some surface areas—likely to be traces of metallic impurities generating delocalized charge states^15^,^33^—that seem to be absent on the AFM images (see also Supplementary Figs 2 and 3).

**Figure 6:**
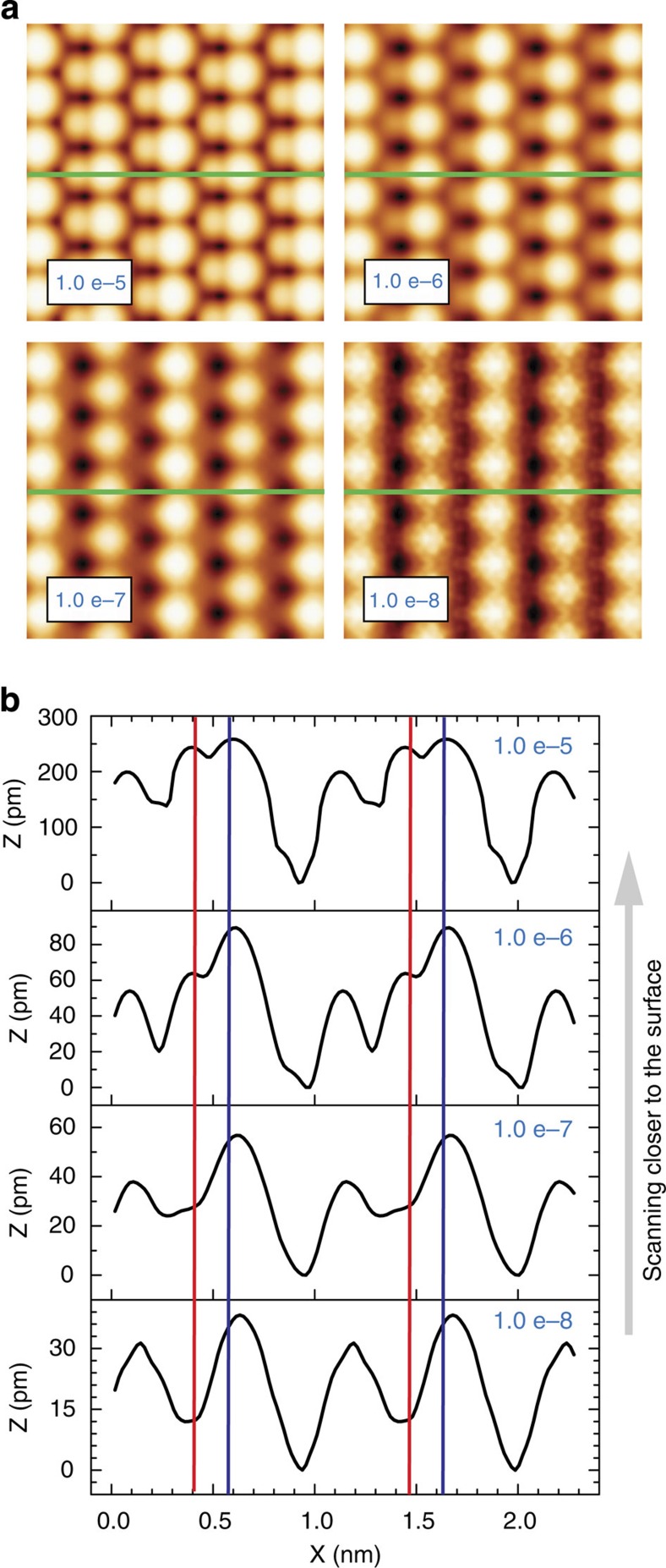
Distance dependence of the STM topography on the TiO_2_ (101) anatase surface. (**a**) Images of the clean surface calculated using the Tersoff-Hamann theory with the one of the large positive bias voltages used in our experiments (+0.8 eV). These images correspond to different isosurfaces (10^−5^ to 10^−8^ e bohr^−3^) of the local density of states of the anatase (101) surface, integrated in a 0.8 eV energy window from the conduction band minimum. Larger isosurface values correspond to STM scans closer to the surface as indicated by the arrow. (**b**) Line profiles corresponding to the green lines in the images and revealing the dependence of the STM topographic corrugation and the relative contribution of the different chemical species with the tip-surface separation distance. The blue (red) vertical lines correspond to the position of the Ti_5*c*_ (O_2*c*_) atoms.

We now consider the relation between the <I_*t*_> and the topographic STM images of the anatase (101) surface. A direct comparison between these two types of images is in principle difficult due to the different acquisition scheme^31^. However, we provide theoretical evidence that makes our <I_*t*_> measurements and previous STM observations compatible^13^,^14^,^15^,^16^,^17^,^18^,^19^,^20^,^33^,^34^,^42^ under a common framework. Figure 6a shows simulated STM images of the clean surface computed with one of the large positive bias voltages used in our experiments (+0.8 V). These calculated images correspond to different isosurfaces of the local density of states of the anatase (101) surface integrated in a 0.8 eV energy window from the conduction band minimum. Larger isosurface values correspond to larger current set points, and thus, to scanning closer to the surface. The images reveal that when scanning with STM at distances far from the surface, the current maxima are essentially spherical and centered around the Ti_5*c*_ atoms, but when scanning at higher set points, the current maxima widen out due to an increasing contribution from the O_2*c*_ atoms. The contribution of the different atomic species to the STM tunnelling current is available from the corresponding line profiles (green line in the images), which are compared in Fig. 6b. The simulated topography corrugation depends strongly on the tip–surface distance. The result closest to our experiments (isosurface of 10^−7^ e bohr^−3^) is associated with a STM imaging mode dominated by the Ti_5*c*_ atoms. The latter corresponds to <I_*t*_> images acquired typically between 4 to 5 Å above the surface, with deconvoluted tunnelling current values^31^ of a few tenths of a nanoampere (see Supplementary Fig. 1). Previous conventional STM measurements and theoretical results^45^ indicate a significant contribution of the O_2*c*_ atoms to the images, which we also obtain for simulated STM images close to the surface. Those theoretical STM images^45^ were calculated at a close distance (2.5 Å) above the surface but with a bias of 1.5 eV, imitating the experimental bias voltages used in conventional STM imaging of this surface. The larger bias voltage also enhances the role of the O_2*c*_ atoms, as it is shown in the projected density of states of the surface atoms (see Supplemental Fig. 5) in which the O_2*c*_ contribution is negligible near the minimum of the conduction band but increases for energies above +0.6 eV. This small contribution of the O_2*c*_ atoms to the local density of states at the bias voltages used in our experiments enhances the contribution of the Ti_5*c*_ atoms to our <I_*t*_> images even when scanning relatively close to the surface.

In summary, our results demonstrate the benefits of combined AFM and averaged tunnelling current imaging for the study of oxide surfaces at the atomic scale, and specially for the characterization of defects and adsorbates that play a crucial role in the catalytic and energy-harvesting applications of these materials. We have applied these techniques for the discrimination and simultaneous imaging of different atomic species—with O_2*c*_ (Ti_5*c*_) dominating the AFM (averaged tunnelling current) channel—at the TiO_2_(101) anatase surface and showed tunnelling current acquisition within the surface band gap, where standard STM imaging is challenging. First-principles simulations provide an explanation for the small tip-surface interactions in terms of an hydroxyl-terminated tip and reveal the role of dynamic tip relaxation effects in contrast formation during AFM imaging. Assisted by key differentiating traits extracted from our first-principles AFM and STM calculations, we have identified candidates for the most common surface defects, such as subsurface oxygen vacancies and surface hydroxyls. Our STM calculations also shed light on the role of the tip–surface separation and the bias voltage on the tunnelling current detected over the anatase (101) surface, explaining the contrast observed in the averaged tunnelling current images with respect to conventional topographic STM images previously reported. The findings reported here provide the foundation for future work on anatase, including the thorough characterization of a large amount of purposefully created defects. More importantly, they pave the way for the study of more complex anatase systems related to water splitting and organic photovoltaics (solar energy conversion), including the adsorption geometries and binding sites of organic dyes and other photoactive molecules like pentacene and buckminsterfullerene, as well as metal dopants to enhance hydrogen production.

## Methods

### Scanning probe microscopy measurements

A UNISOKU Ltd ultrahigh vacuum (UHV) cryogenic dynamic AFM with a home-built optical interferometer for the detection of the cantilever dynamics and a commercial scanning probe microscopy (SPM) controller (Nanonis SPM Control System, SPECS, Germany) was used for the experiments and the *in situ* sample preparation. Measurements were accomplished at a 77 K tip-sample temperature using frequency modulation detection^46^. Platinum–iridium-coated silicon cantilevers (PPP-NCLPt-20, Nanosensors, Switzerland) were instantaneously excited to their first mechanical resonant frequency keeping the oscillation amplitude constant. The tunnelling current flowing between probe and surface averaged over multiple cantilever oscillation cycles (<I_*t*_>)^31^ was simultaneously detected during topographic AFM imaging (see also the discussion in Supplementary Fig. 3). The shift of the first mechanical resonant frequency (Δ*f*) from the free-oscillation value on forces acting on the cantilever probe was used to regulate the tip–surface separation.

The abbreviations for the experimental parameters are as follows: *f*_o_ is the free-oscillation fundamental resonant frequency of the cantilever; Δ*f* is the frequency shift set point for AFM topographic imaging; *A* is the cantilever oscillation amplitude; *K* is the cantilever stiffness; CPD is the tip–surface contact potential difference measured a few nanometers above the surface plane; *V*_Bias_ is the bias voltage applied to the sample.

Force spectroscopy^47^ was carried out by recording both Δ*f* and <I_*t*_> as a function of the tip-sample relative vertical displacement (*Z*). The determination of the corresponding *K* and *A* values are described elsewhere^48^. The absence of any tip or surface modification during the spectroscopic acquisition was carefully checked^48^. In standard AFM imaging and force spectroscopic measurements, the long-range electrostatic interaction was minimized by compensating the CPD. For the characterization of the <I_*t*_> signal a small *V*_Bias_ was applied during both imaging and force spectroscopy acquisition. The total tip–surface interaction force was obtained from Δ*f*(*Z*) curves by applying an inversion procedure^49^, and the <I_*t*_> signal was related to the expected static STM values (Supplementary Fig. 1) by implementing the corresponding conversion method^31^. Topographic effects on *Z* due to the spectroscopic acquisition were compensated^50^, assuring a common origin for curves measured at different locations with respect to the surface plane. The tip–surface interatomic forces were obtained by subtracting an appropriate fit^47^,^48^ over the long-range interaction region to the total force. For the comparison of the experimental and theoretical forces in Fig. 3, the experimental curves were shifted—keeping constant their relative separation—a distance that aligns the position of the minimum for the O_2*c*_ curve with the theoretical counterpart.

Bias-dependent imaging was carried out by opening the feedback loop for the short time required to change the bias voltage and update the Δ*f* topographic set point to the value read after applying the new bias potential. This Δ*f* set point accounts for the additional electrostatic force with respect to the CPD compensated case. This method prevents a slight tip retraction from the surface on increasing the electrostatic force, approximately keeping the same tip–surface separation for images acquired at different bias voltages.

Prior to the measurements, the cantilever tip was conditioned for simultaneous AFM and averaged tunnelling current acquisition by performing current-bias spectroscopy in static STM mode while approaching the tip towards the surface several ångströms. This procedure often results in the tip picking up surface material—holes of a few nanometers diameter were normally detected at the surface after the tip conditioning—in an environment where most of the UHV residual gas consists of hydrogen molecules.

### Sample preparation

Natural single crystals of anatase cut exhibiting a polished (101) surface were purchased from Surface Net GmbH (www.surfacenet.de). The surface preparation was made by successive cycles of 20 min. Ar^+^ ion sputtering (1 keV energy and 5 μA ion current measured at the sample) and annealing at 970 K during 30 min in UHV (∼1.2 × 10^−8^ Pa). The anatase natural crystals used in this work have a variety of impurities that may differ in concentration and nature from one crystal to another. With our sample mounting and surface preparation protocol, we have not observed the formation of surface structures assigned to segregation of impurities^51^. Water dosage was carried out by exposing a clean anatase surface to 0.8 Langmuir of water vapour while keeping the sample cold. Ultra-clean Milli-Q water—further purified by several freeze–pump–thaw cycles—was introduced into the UHV system as vapour via a leak valve.

### First-principles calculations

Calculations based on density functional theory were performed using the VASP code^52^, with PAW pseudopotentials and a plane wave basis set with a cutoff of 500 eV. The PBE exchange-correlation functional^53^ was supplemented by onsite *U*=4 eV terms on Ti atoms to describe better the electronic structure of the TiO_2_(101) anatase surface and the electron localization on 
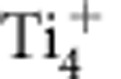
 sites associated with the creation of its most common point defects. This *U* value is compatible with recent theoretical results^33^. The surface was modelled as a 10.57 × 11.56 Å periodic slab of four TiO_2_ trilayers, with the bottom two layers fixed into a bulk-like configuration. Optimized structures were obtained with Γ point sampling of the Brillouin zone. Further surface electronic structure properties, including Tersoff–Hamann STM images^54^, were extracted from static calculations featuring a 4 × 4 Monkhorst–Pack k-point mesh.

The tip–surface interaction energy and force were determined in a stepwise, quasistatic manner by lowering the tip model towards the surface in steps of 25 pm from the original height of 6 Å. At each step, the atoms in the top two slab trilayers and the bottom of the tip model were allowed to relax into their ground state configuration with convergence criteria for the total energy and forces of 10^−6^ eV and 0.01 eV Å^−1^. Smooth force curves were obtained by fitting a Morse potential function modified by a polynomial to the theoretical force data points.

## Additional information

**How to cite this article:** Stetsovych, O. *et al.* Atomic species identification at the (101) anatase surface by simultaneous scanning tunnelling and atomic force microscopy. *Nat. Commun.* 6:7265 doi: 10.1038/ncomms8265 (2015).

## Supplementary Material

Supplementary Information Supplementary Figures 1-5
