# Supplementary material for: Atomic species identification at the (101) anatase surface by simultaneous scanning tunnelling and atomic force microscopy
Source: Nat Commun. 2015 Jun 29;6:7265. doi: 10.1038/ncomms8265 (PMC4491188; doi:10.1038/ncomms8265)
Supplement: Supplementary Information — Supplementary Figures 1-5 [file ncomms8265-s1.pdf]

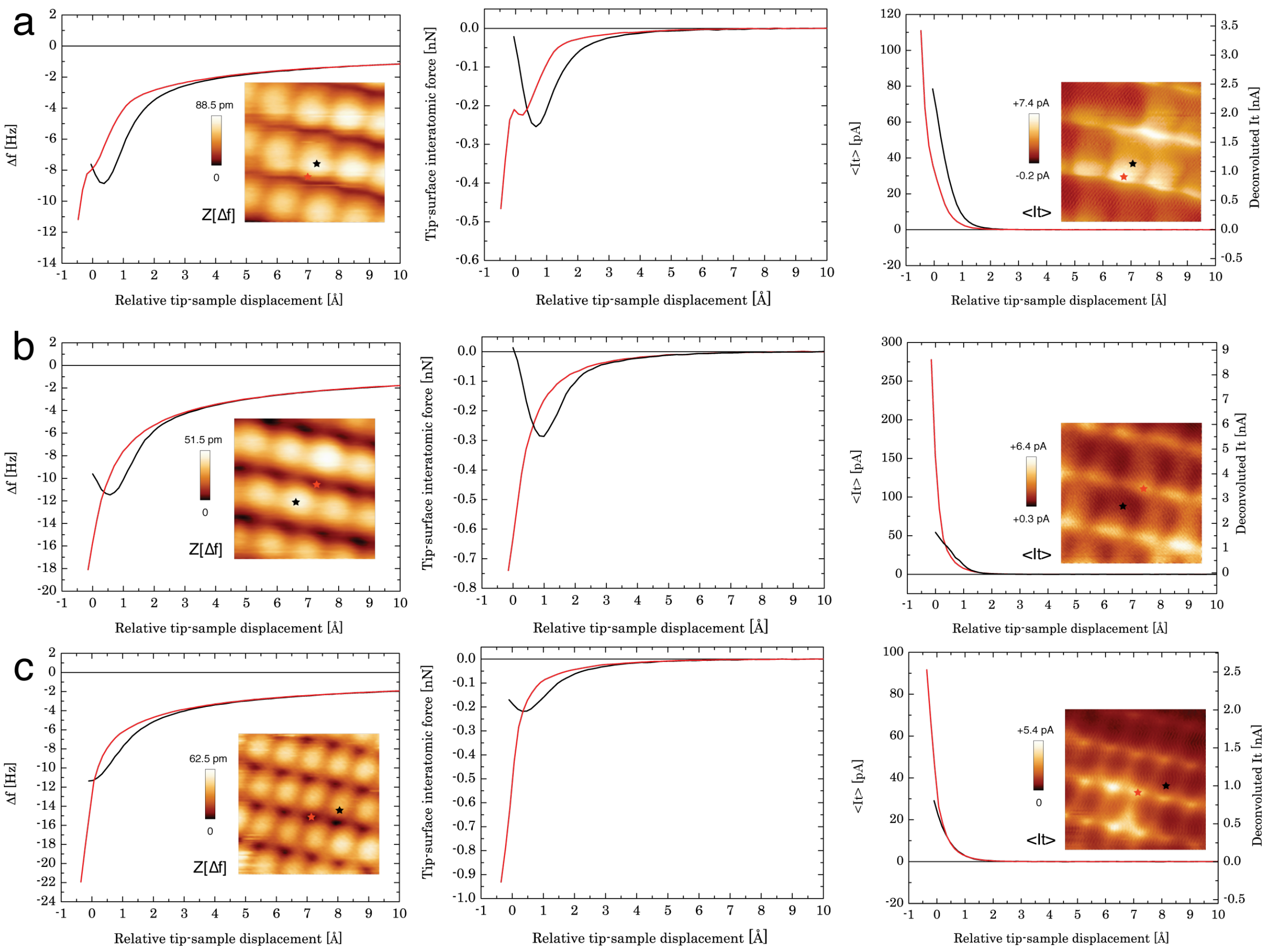

Supplementary Figure 1: Force spectroscopy measurements on the TiO_2 anatase surface. **a**, **b** and **c**, Variation of the frequency shift (left), the tip-surface interatomic force (centre) and the average tunnelling current (right) as a function of the tip-surface relative displacement upon approaching the AFM probe towards the surface. The corresponding tunnelling current values after deconvolution of the tip oscillation effect on the average tunnelling current are shown on the right axis of the graph. For acquisition details and calculation of the experimental tip-surface interatomic forces see the *Methods* section in the main text. The insets show simultaneous AFM topographic (Z[Δf]) and average tunnelling current ($\langle I_t \rangle$) images of the surface area where the spectroscopic measurements were performed. The black and red stars within the images pinpoint the exact location where the curves were acquired. These spectroscopy series were measured with the same cantilever but three different tip apexes upon conditioning the cantilever tip for atomic resolution imaging. Image dimensions are (1.5 × 1.5) nm^2 for (a) and (b), and (2 × 2) nm^2 for (c). Acquisition parameters are: f_o= 160360 Hz, Δf = -5.0 Hz, A= 113.6 Å, K= 27.5 $N{\cdot}m^{-1}$, CPD= -80 mV, V_{Bias}= +500 mV for (a); f_o= 160358 Hz, Δf = -6.0 Hz, A= 116.1 Å, K= 27.5 $N{\cdot}m^{-1}$, CPD= -150 mV, V_{Bias}= +500 mV for (b); and f_o= 160366 Hz, Δf = -7.5 Hz, A= 102.2 Å, K= 27.5 $N{\cdot}m^{-1}$, CPD= -75 mV, V_{Bias}= +500 mV for (c). During the acquisition of these spectroscopy measurements, the bias voltage was kept at the same value as for imaging to study the dependence of the $\langle I_t \rangle$ signal with the tip-surface separation. The origin in the relative tip-sample displacement is the point of closest approach of the tip towards the surface at the O_{2c} site.

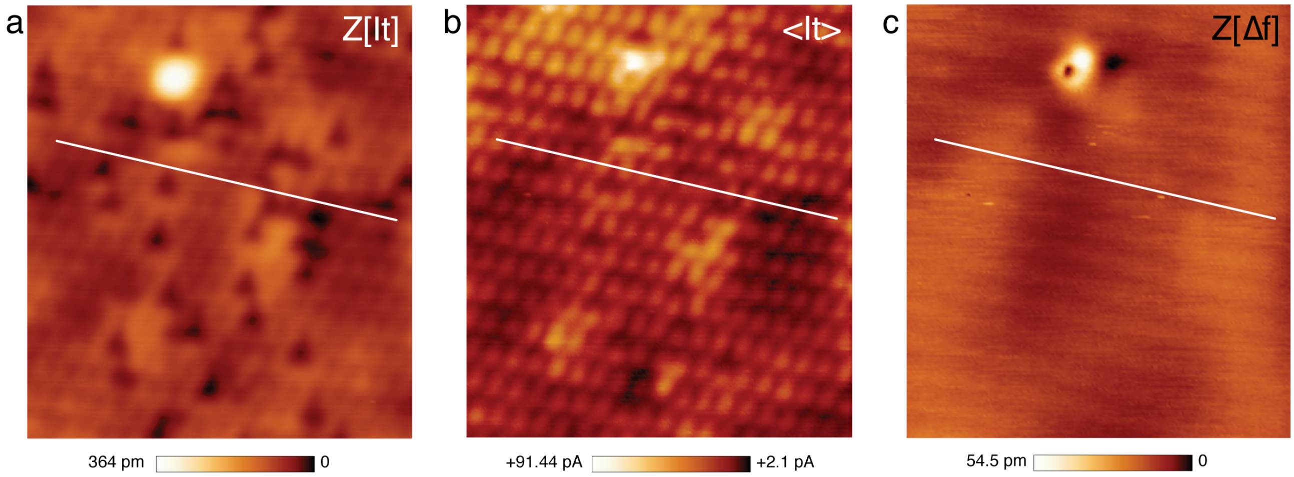

Supplementary Figure 2: Standard STM imaging with platinum-iridium coated silicon cantilevers. a, STM image of the TiO_2(101) anatase surface. This image was acquired during a simultaneous dynamic AFM-STM measurement session by suppressing the cantilever oscillation, setting the tunnelling current as control signal for the topographic feedback (Z[I_t]), and by applying a relatively high sample bias voltage. **b** and **c**, Simultaneous averaged current tunnelling (<I_t>) and topographic AFM (Z[Δf]) images of the same surface area. The line on top of the images approximately marks the same location at the surface. Since the images in (a) and (b) were not simultaneously recorded, it is difficult to establish a precise correspondence between the protrusions detected in Z[I_t] and in <I_t>. However, a comparison of both pictures suggests that the same surface atomic row is imaged in both of them. The AFM topography in (c) has a faint resolution because the feedback set point was adjusted to a value where the tip-surface interatomic forces are barely sensed. Nonetheless, the surface region highlighted by the line confirms the general trend detected in the simultaneous dynamic AFM-STM images presented in this work: the <I_t> maxima lie in between the rows of protrusions observed in the topographic AFM image (see also Supplementary Figure 3 and Fig. 1 in the main text). Image dimensions are (8.6 × 9.7) nm^2. Acquisition parameters are: I_t = 0.1 nA, V_{Bias}= +1.4 V for (a); and f_o= 153031 Hz, Δf = -3.5 Hz, A= 107.1 Å, K= 23.9 N·m^{-1}, CPD= V_{Bias}= +740 mV for (b) and (c).

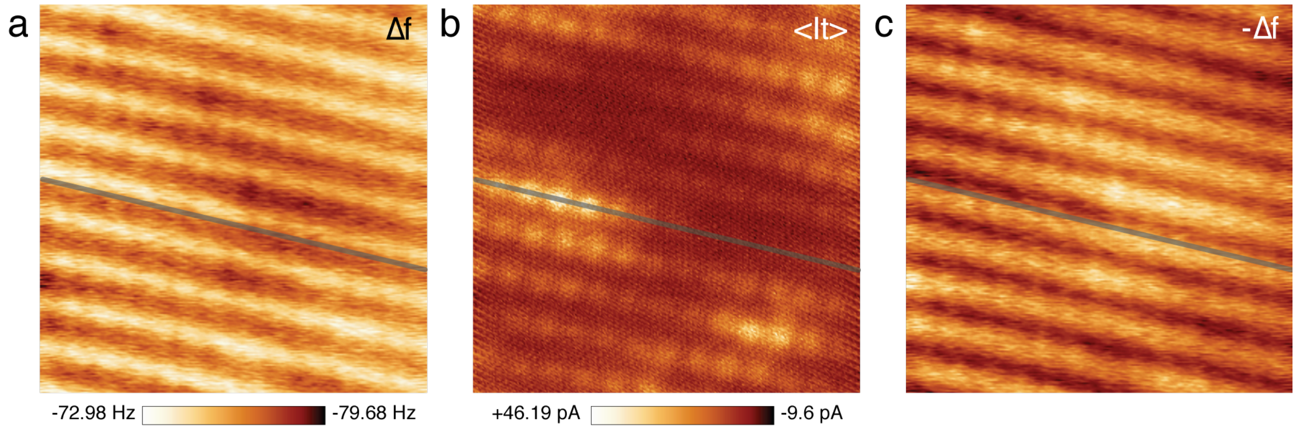

Supplementary Figure 3: Constant height imaging. **a** and **b**, Simultaneous AFM (Δf) and averaged tunnelling current (<I_t>) images measured over the TiO_2(101) anatase surface scanning in constant height mode. For the acquisition of these data, the topographic AFM feedback loop was open and the surface area was scanned at high speed to avoid the variation of the tip-sample separation —caused by thermal drift— during the time interval the image was obtained (the noisy appearance of the images is due to a high speed scan). **c**, Panel displayed in (a) with inverted contrast for a better comparison with topographic AFM images. The lines mark the same location on the three images. The comparison of these data suggests that the Δf and the <I_t> signals are spatially decoupled when imaging the TiO_2(101) anatase surface with atomic resolution. This feature grants a negligible modulation of the <I_t> signal when scanning the surface using topographic AFM feedback. In general, for systems where the tunnelling current and the tip-surface interaction force are not spatially decoupled, <I_t> acquisition with topographic AFM feedback is not fully reliable for an independent interpretation of both data channels, and constant (or quasi-constant) height scanning is mandatory. The surface region highlighted by the line confirms again that the <I_t> maxima lie in between the rows of higher tip-surface interatomic forces. Image dimensions are (5 × 5) nm^2. Acquisition parameters are: f_o= 151937 Hz, A= 74.7 Å, K= 23.4 $N \cdot m^{-1}$, CPD= -650 mV, V_{Bias}= +400 mV.

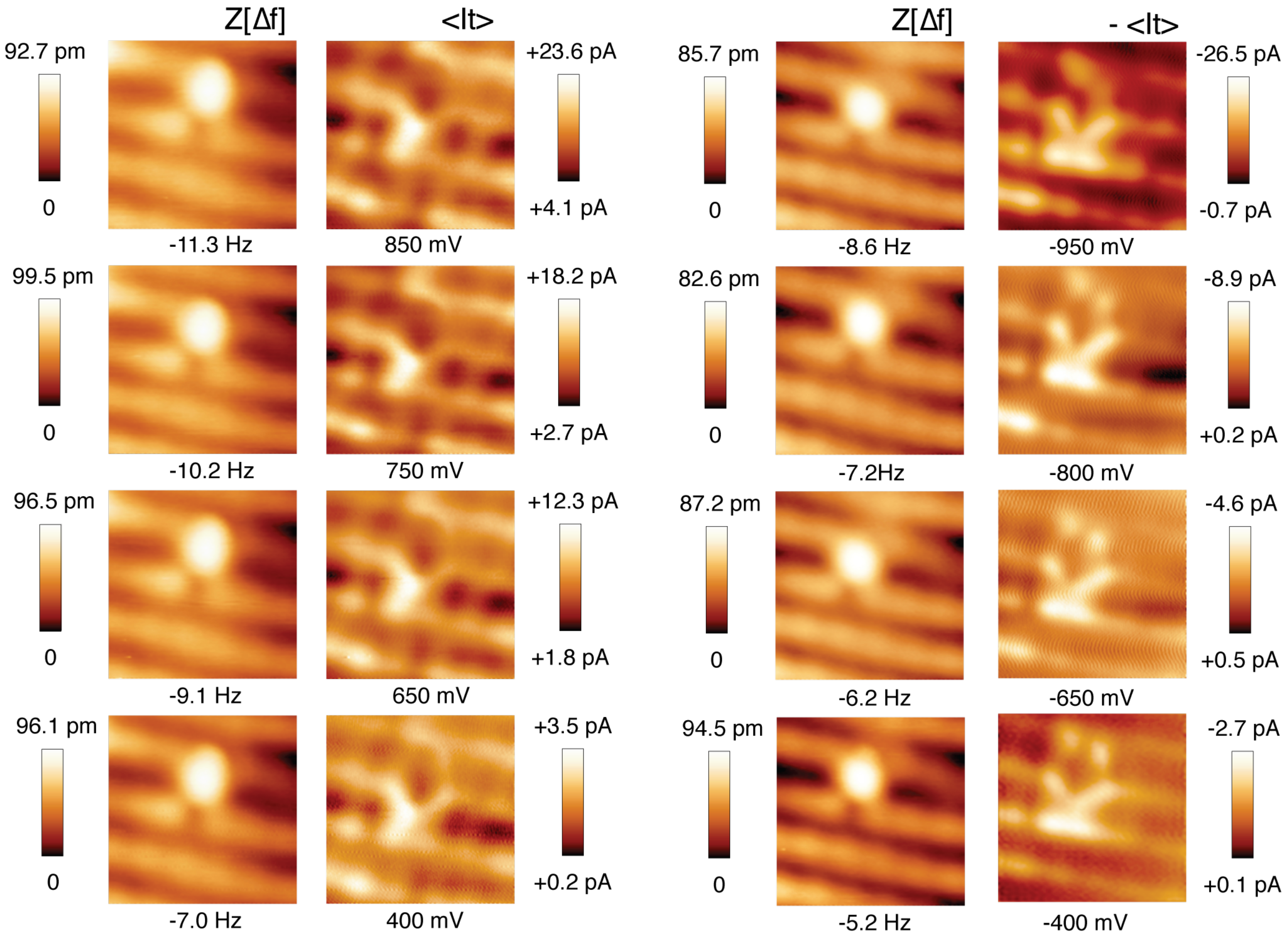

Supplementary Figure 4: Figure 2e is presented here without the atomic model of the anatase (101) surface superimposed to the images for a better appraisal of the features exhibited by the simultaneous AFM topography (Z[Δf]) and averaged tunnelling current (<I_t>) signals. Image dimensions and acquisition parameters can be found in the caption of figure 2e in the main text.

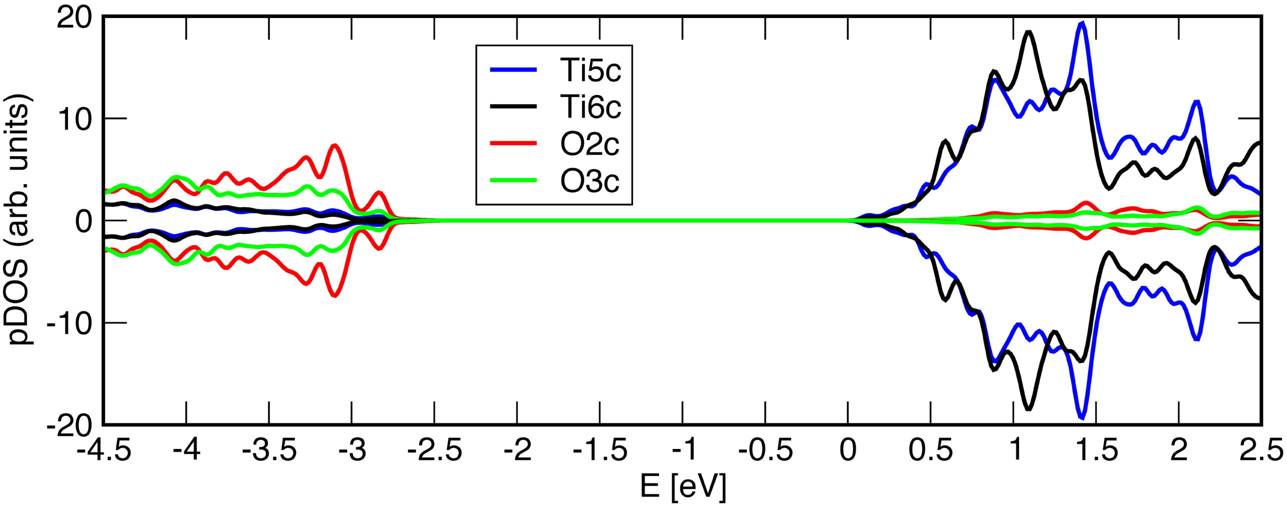

Supplementary Figure 5: Projected density of states (pDOS) around the anatase energy gap for the relevant atomic species populating the surface. The energy zero corresponds to the minimum of the conduction band (CBM). The contribution of the O2c atoms is negligible for energies close to the CBM but increases significantly for energies above 0.6 eV.
